# Supplementary material for: Flower diversity and bee reproduction in an arid ecosystem
Source: PeerJ. 2016 Jul 26;4:e2250. doi: 10.7717/peerj.2250 (PMC4974926; doi:10.7717/peerj.2250)
Supplement: Supplemental Information 1 [file peerj-04-2250-s001.doc]

Table S1. Geographic location, altitude, post-fire age, flower density, and flower richness of the study sites.

| Latitude | Longitude | Altitude | Years from last fire * | Flower density | Rarefied flower richness |
| --- | --- | --- | --- | --- | --- |
| 32º32'19.4” S | 68º57'18.4” WO | 1259 | 8 | 48850 | 12.79 |
| 32º31'50.7” S | 68º56'52.1” WO | 1225 | 8 | 118016 | 13.50 |
| 32º33'49.8” S | 68º57'01.8” WO | 1239 | 2 | 79547 | 19.44 |
| 32º31'23.3” S | 68º56'22.6” WO | 1235 | >25 | 118016 | 14.30 |
| 32º30'26.3” S | 68º56'14.5” WO | 1238 | >25 | 157905 | 15.50 |
| 32º31'57.6” S | 68º59'08.72” WO | 1450 | 22 | 82580 | 15.63 |
| 32º30'26.3” S | 68º58'42.1” WO | 1383 | 1 | 72320 | 15.04 |
| 32º28'39.6” S | 68º56'21.8” WO | 1398 | >25 | 43169 | 13.53 |
| 32º30'01.4” S | 68º55'20.7” WO | 1174 | >25 | 58547 | 15.67 |
| 32º33'55.3” S | 68º56'03.7” WO | 1151 | 8 | 226526 | 10.89 |
| 32º36'09.3” S | 68º58'08.6” WO | 1208 | 17 | 104528 | 13.99 |
| 32º35'01.4” S | 68º58'11.2” WO | 1269 | 17 | 137823 | 15.96 |
| 32º34'42.9” S | 68º57'01.8” WO | 1182 | 7 | 88504 | 15.88 |
| 32º35'40.0” S | 68º55'33.5” WO | 1078 | >25 | 167615 | 8.64 |

* Post-fire age estimated in 2008 (E.L. Stevani, personal communication).
