# Supplementary material for: Flower diversity and bee reproduction in an arid ecosystem
Source: PeerJ. 2016 Jul 26;4:e2250. doi: 10.7717/peerj.2250 (PMC4974926; doi:10.7717/peerj.2250)
Supplement: Supplemental Information 2 — The variables in brackets are the independent ones and variables in {} are the conditional ones. [file peerj-04-2250-s002.docx]

Table S2. Basis set used for d-sep test used to evaluate the goodness of fit of the models in Figure “model 1 and 2”. The variables in braquets are the independent ones and variables in {} are the conditional ones.

| Model | Basis set |
| --- | --- |
| 1 | (Altitude, Time elapsed since last fire)\|{Ǿ},  (Altitude, Temporal stability in flower production)\|{Flower richness},  (Flower richness, Flower abundance)\|{Altitude},  (Flower abundance, Temporal stability in flower production)\|{Flower richness, Altitude},  (Time elapsed since last fire, Flower abundance)\|{Altitude},  (Time elapsed since last fire, Flower ricness)\|{Altitude},  (Time elapsed since last fire, Temporal stability in flower production)\|{Flower richness}, |
| 2 | (Altitude, Temporal stability in flower production)\| {Flower richness} |
