# Supplementary figures and images for: Flower diversity and bee reproduction in an arid ecosystem
Source: PeerJ. 2016 Jul 26;4:e2250. doi: 10.7717/peerj.2250 (PMC4974926; doi:10.7717/peerj.2250)

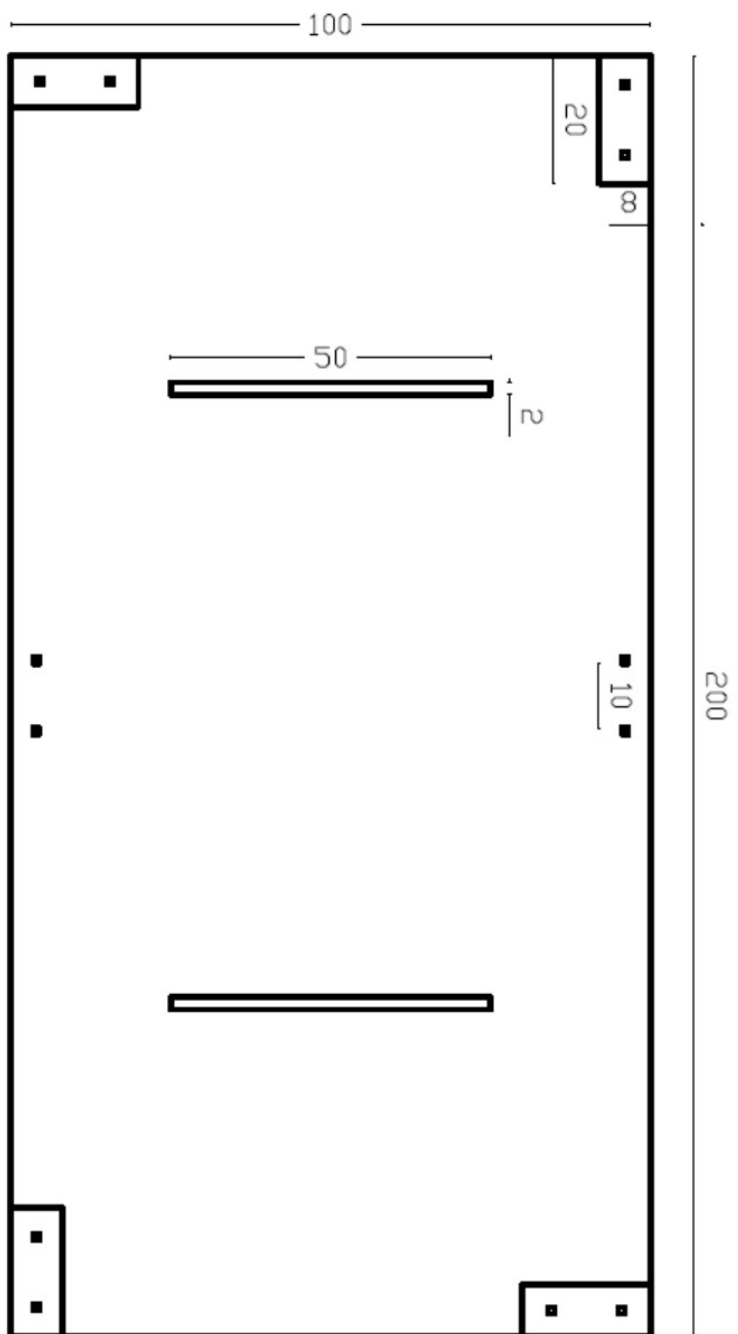

Supplement: Supplemental Information 3 [file peerj-04-2250-s003.pdf]

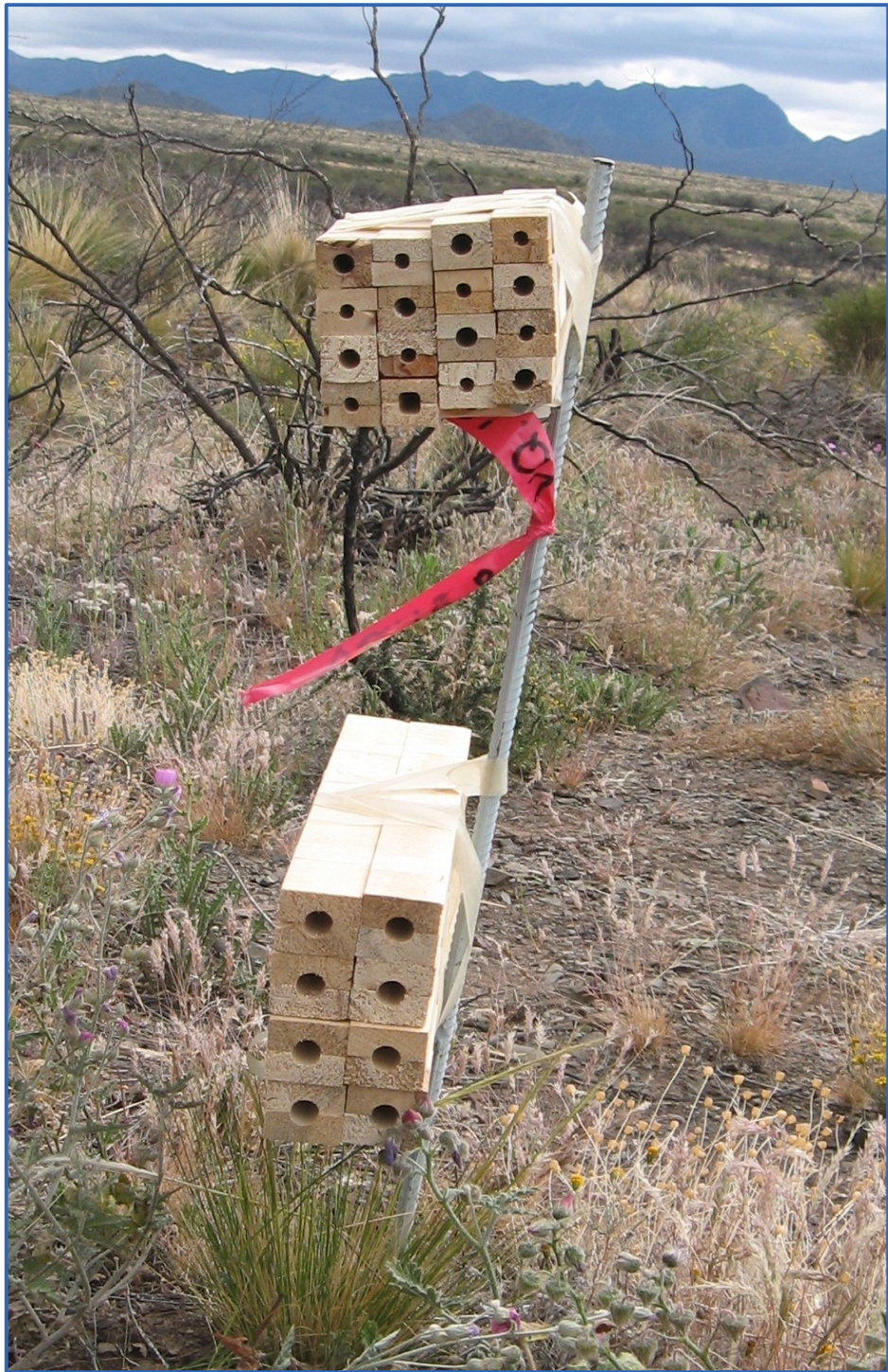

Supplement: Supplemental Information 4 — Trap nests consist of wood pieces with a longitudinal hole of three different diameter where bee species nest. Each occupied trap nest constitutes one bee nest. [file peerj-04-2250-s004.pdf]
