# Supplementary material for: Flower diversity and bee reproduction in an arid ecosystem
Source: PeerJ. 2016 Jul 26;4:e2250. doi: 10.7717/peerj.2250 (PMC4974926; doi:10.7717/peerj.2250)
Supplement: Supplemental Information 5 — Model 1 describes the effect of flower diversity (estimated using flower richness), flower abundance (estimated using flower density) and temporal stability of flower production along the flowering season (estimated as the inverse of coefficient of variation of the weekly flower abundance mean), fire (estimated as time elapsed since last fire), and elevation (estimated as meters above the sea). Y axis represents the path coefficients that rank from 1 to −1, and the x axis represents the effect of above mentioned ecological variables on three bee reproductive variables: A is the average brood cell number per nest per site, B is the total number of brood cells per site, and C is the total number of nests per site. In blue color are the ninety-five percent confidence limits of path coefficients obtained from bootstrap sampling of the distribution of path coefficients. [file peerj-04-2250-s005.pdf]

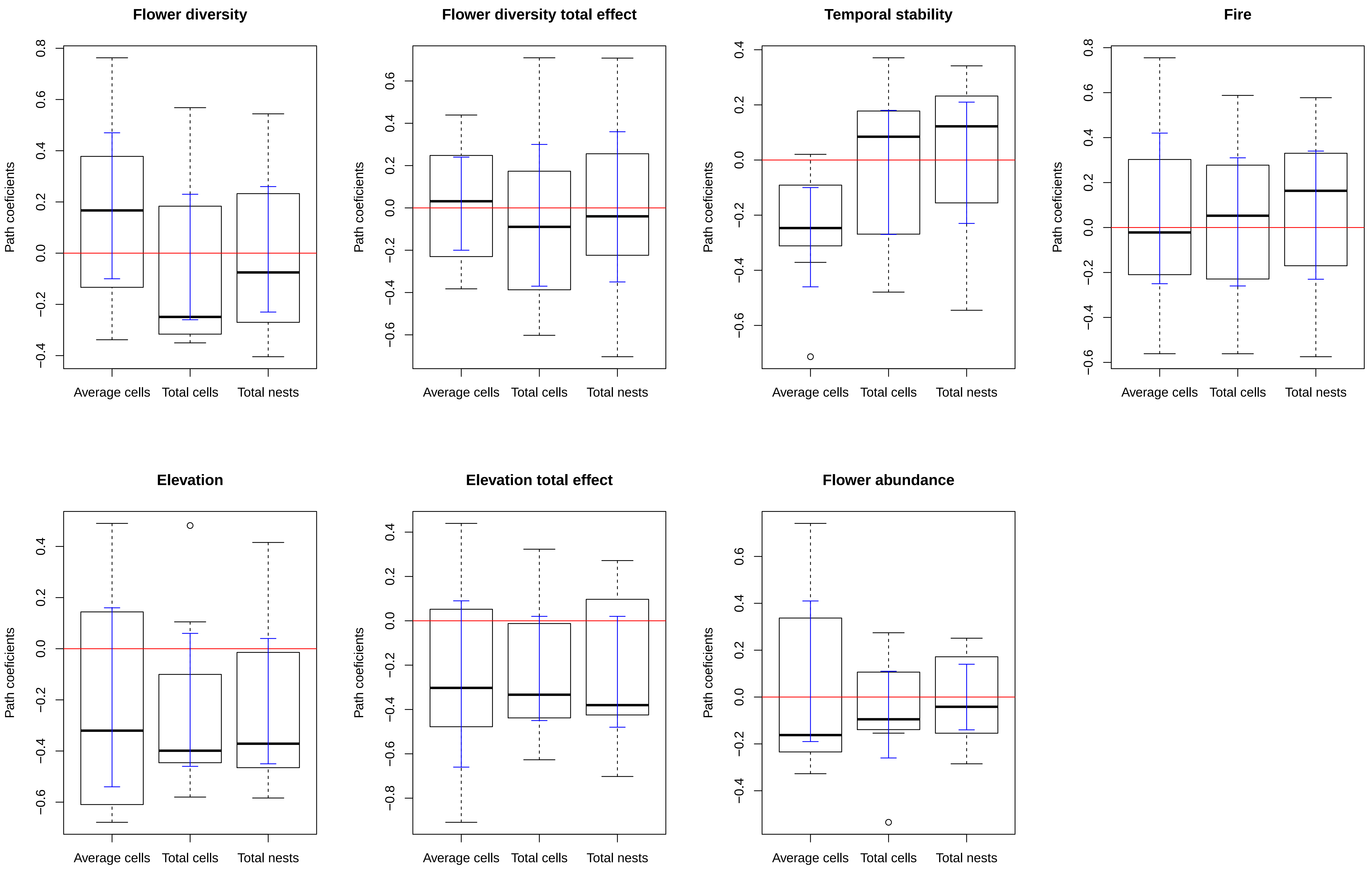
Flower diversity
Path coeficients
0.8
0.6
0.4
0.2
0.0
−0.2
−0.4
Average cells
Total cells
Total nests
Flower diversity total effect
Path coeficients
0.6
0.4
0.2
0.0
−0.2
−0.4
−0.6
Average cells
Total cells
Total nests
Temporal stability
Path coeficients
0.4
0.2
0.0
−0.2
−0.4
−0.6
Average cells
Total cells
Total nests
Fire
Path coeficients
0.8
0.6
0.4
0.2
0.0
−0.2
−0.4
−0.6
Average cells
Total cells
Total nests
Elevation
Path coeficients
0.4
0.2
0.0
−0.2
−0.4
−0.6
Average cells
Total cells
Total nests
Elevation total effect
Path coeficients
0.4
0.2
0.0
−0.2
−0.4
−0.6
−0.8
Average cells
Total cells
Total nests
Flower abundance
Path coeficients
0.6
0.4
0.2
0.0
−0.2
−0.4
Average cells
Total cells
Total nests
